# Resveratrol Ameliorates Microcystin-LR-Induced Testis Germ Cell Apoptosis in Rats via SIRT1 Signaling Pathway Activation

**DOI:** 10.3390/toxins10060235

**Published:** 2018-06-09

**Authors:** Haohao Liu, Shenshen Zhang, Chuanrui Liu, Jinxia Wu, Yueqin Wang, Le Yuan, Xingde Du, Rui Wang, Phelisters Wegesa Marwa, Donggang Zhuang, Xuemin Cheng, Huizhen Zhang

**Affiliations:** College of Public Health, Zhengzhou University, Zhengzhou 450001, China; Liuhlin2018@126.com (H.L.); zsslb2005@163.com (S.Z.); lcr0624@126.com (C.L.); wjxsir@126.com (J.W.); wyq2018@stu.zzu.edu.cn (Y.W.); yl19920215@126.com (L.Y.); dxd1993@163.com (X.D.); wr935314032@163.com (R.W.); wegesalee@gmail.com (P.W.M.); zdg@zzu.edu.cn (D.Z.); cxm@zzu.edu.cn (X.C.)

**Keywords:** apoptosis, microcystin-LR (MC-LR), reproductive toxicity, resveratrol, sirtuin 1 (SIRT1)

## Abstract

Microcystin-leucine arginine (MC-LR), a cyclic heptapeptide produced by cyanobacteria, is a strong reproductive toxin. Studies performed in rat Sertoli cells and Chinese hamster ovary cells have demonstrated typical apoptosis after MC-LR exposure. However, little is known on how to protect against the reproductive toxicity induced by MC-LR. The present study aimed to explore the possible molecular mechanism underlying the anti-apoptosis and protective effects of resveratrol (RES) on the co-culture of Sertoli–germ cells and rat testes. The results demonstrated that MC-LR treatment inhibited the proliferation of Sertoli–germ cells and induced apoptosis. Furthermore, sirtuin 1 (SIRT1) and Bcl-2 were inhibited, while p53 and Ku70 acetylation, Bax expression, and cleaved caspase-3 were upregulated by MC-LR. However, RES pretreatment ameliorated MC-LR-induced apoptosis and SIRT1 inhibition, and downregulated the MC-LR-induced increase in p53 and Ku70 acetylation, Bax expression, and caspase-3 activation. In addition, RES reversed the MC-LR-mediated reduction in Ku70 binding to Bax. The present study indicated that the administration of RES could ameliorate MC-LR-induced Sertoli–germ cell apoptosis and protect against reproductive toxicity in rats by stimulating the SIRT1/p53 pathway, suppressing p53 and Ku70 acetylation and enhancing the binding of Ku70 to Bax.

## 1. Introduction

Cyanobacterial blooms caused by water eutrophication represent a health hazard to humans and animals, evoking global concerns [1,2]. Microcystins (MCs) are a family of over 100 different structural analogue compounds with seven stable cyclic heptapeptide structures, and are produced by cyanobacteria such as *Microcystis* [3]. Microcystin-leucine arginine (MC-LR) is the most abundant and most toxic MC found in natural water, causing growing environmental and public health issues [4]. Humans are most likely exposed to MC-LR through the consumption of contaminated water and food resources, and dermal exposure/inhalation during recreational activities in contaminated surface water. Thus, a safety limit (1.0 μg/L) of MC-LR has been set by World Health Organization (WHO) in drinking water. However, the concentration is usually much higher in natural water. Chen et al. considered that further studies are needed to determine whether the present WHO provisional MC-LR guideline for drinking water is protective for humans [5]. 

MC-LR can accumulate in several tissues such as the liver, brain, ovary, intestine, kidney, and muscle [6,7,8,9,10]. The liver is the most affected organ in humans, followed by the gonads [11]. Accordingly, MC-LR has been shown to induce sperm abnormalities by downregulating miR-96 and altering deleted-in azoospermia-associated protein 2 (DAZAP2) expressions [12]. Chen et al. found that MC-LR was cytotoxic to Sertoli cells by altering the expression of miRNAs and mRNAs [13]. In a previous study conducted by the investigators, it was demonstrated that Chinese hamster ovary (CHO) cell apoptosis after MC-LR treatment may be associated with the activation of endoplasmic reticulum stress (ERs) and autophagy [14]. 

Sirtuin 1, which is a member of the sirtuin family of proteins encoded by the *SIRT1* gene and is also a NAD-dependent deacetylase protein [15], is associated with the regulatory control of diverse cellular process including cell survival, apoptosis, DNA repair, autophagy, and cell migration, through deacetylating histones and non-histones proteins [16,17]. SIRT1 could regulate p53 activity through deacetylation modification [18]. Acetylation plays a vital role in the activation of p53. Acetylated p53 induces the expression of many genes, causing either cell cycle arrest or apoptosis [19]. The study conducted by Vaziri et al. [18] demonstrated that SIRT1 downregulated the acetylated p53 levels, reduced *p53* transcriptional activity, and prevented p53-dependent apoptosis. P53 is a central stress sensor that responds to apoptosis, cell death, oxidative stress, and autophagy, which can stimulate the expression of Bax and suppress Bcl-2 protein expression, and thereby induce apoptosis through the mitochondria-dependent pathway [20,21]. Recent studies showed that the enhanced expression of SIRT1 could decrease p53 acetylation, thereby inhibiting mitochondria apoptosis [22,23]. Similarly, the potent SIRT1 activator resveratrol (RES) enhances cell survival and inhibits apoptosis by stimulating SIRT1 activation and the deacetylation of p53 [17,24,25].

Ku70, a key factor of the non-homologous end joining (NHEJ), is one of the crucial downstream mediators of SIRT1. It is an evolutionarily conserved protein that regulates cell death by binding to the proapoptotic factor Bax in the cytoplasm [26]. Cohen et al. have shown that increased acetylation of Ku70 could induce disruption of the Ku70–Bax interaction, which blocks Bax-mediated apoptosis [27]. The acetylation of Ku70 can trigger Bax release and activation, leading to Bax-mediated cell death [28,29]. In addition, the SIRT1 protein can directly interact with Ku70 to physically form a complex that controls the acetylation status of Ku70 protein. Furthermore, Ku70 deacetylation by SIRT1 can promote DNA repair, thereby extending its life span [30,31]. 

Sertoli cells are scaffolds of germ cells that can form a blood–testis barrier through tight junctions, which protect sperm formation and provide a high concentration of androgen environment for sperm maturation. Germ cells acquire nutrients through Sertoli cells, and the structural changes of Sertoli cells play a vital role in the apoptosis of germ cells. In this study, Sertoli cells were used as a feeder layer for germ cells to stimulate the reproductive environment in vivo, and investigate the unexplored SIRT1/p53 pathway-mediated apoptosis. The Sertoli cells and germ cells co-cultured in a model were insufficient in the past single Sertoli cell culture system, but have scientific and practical significance for the study of the reproductive toxicity of MC-LR. 

RES is a potent activator of SIRT1, but little is known about its effects on the acetylation of Ku70 and p53, and eventually, the MC-LR-induced testis germ cell apoptosis. Therefore, the present study was designed: (1) to investigate the expression of SIRT1 and the acetylation of Ku70 and p53 in vitro and in vivo following treatment with MC-LR or RES; (2) explore its related signaling pathway and underlying mechanism; and (3) reveal the effects of RES on MC-LR-induced germ cell apoptosis.

## 2. Results

### 2.1. The Identification and Viability of Co-Cultured Sertoli–Germ Cells

**Figure 1:**
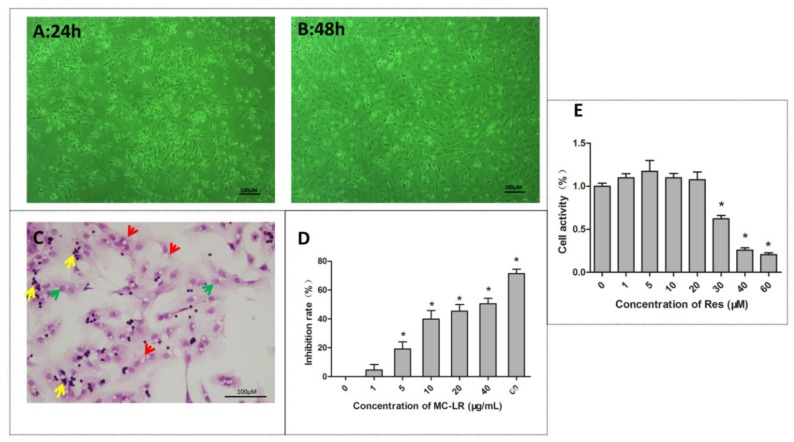
The identification and viability of co-cultured Sertoli–germ cells. Hematoxylin and eosin (H&E) staining was used to identify the co-cultured Sertoli–germ cells. (**A**,**B**) The appearance of co-cultured Sertoli–germ cells after culture for 24 or 48 h by light microscopy (×100); (**C**) H&E staining used to identify the co-cultured Sertoli–germ cells (×200), showing degenerated germ cells (red arrow), Sertoli cell nucleus (green arrow), and germ cells (yellow arrow). (**D**,**E**) The viability of co-cultured Sertoli–germ cells after 24 h of treatment with microcystin-leucine arginine (MC-LR; 0–60 µg/mL) or resveratrol (RES; 0–60 µM) was tested by Cell Counting Kit-8 (CCK8) kits. The calculated IC_50_ dose of MC-LR was 36 μg/mL. The dose used for RES was 20 μM. The results are expressed as mean ± SEM (*n* = 3); * *p* < 0.05 vs. the control group. Scale bar: 100 μm.

Sertoli cells are shuttle-like polygonal membranous epithelioid cells that grow by adhering to the plate, and proliferate in vitro. Germ cells are round and are mainly attached to Sertoli cells for growth. Sertoli cells begin to adhere and germ cells begin to attach to differentiating Sertoli cells after 24 h of incubation (Figure 1A). Sertoli cells flatten out to form a monolayer and germ cell clusters remain attached to the monolayer after 48 h of incubation (Figure 1B). The hematoxylin and eosin (H&E) staining results revealed that there were many vacuoles in the cytoplasm of Sertoli cells, which were degenerated germ cells (red arrow). Furthermore, the Sertoli cell nucleus was large and elliptic (green arrow), while germ cells were round with a deeply stained nuclei (yellow arrow) (Figure 1C). 

Cell Counting Kit-8 (CCK8) kits were used to assess the viability of Sertoli–germ cells exposed to MC-LR or RES for 24 h. As shown in Figure 1D, cell viability gradually decreased with the increase in concentration of MC-LR (1–60 μg/mL). The IC_50_ dose of MC-LR for Sertoli–germ cells was 36 μg/mL by calculating cell inhibition rate. Hence, IC_50_/4, IC_50_/2, and IC_50_ were used for the subsequent experiments. Cell viability slightly increased after treatment with RES (1–20 μM), but declined significantly when the concentration ranged from 30 μM to 60 μM (Figure 1E). Hence, the selected dose of RES in subsequent experiments was 20 μM.

### 2.2. Protective Effect of RES on MC-LR-Induced Testicular Cell Apoptosis

**Figure 2:**
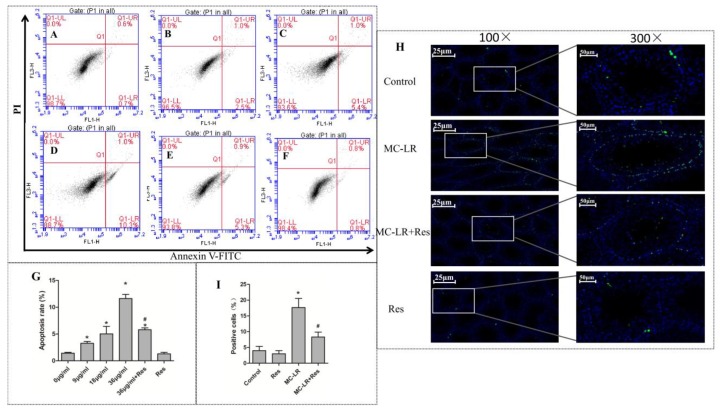
The protective effect of RES on MC-LR-induced testicular cell apoptosis. (**A**–**G**) The apoptotic cells were quantified by flow cytometric analysis. Sertoli–germ cells were stained with fluoresceine isothiocyanate-coupled annexin V and propidium iodide: (**A**) Control; (**B**) 9 μg/mL; (**C**) 18 μg/mL; (**D**) 36 μg/mL; (**E**) RES + 36 μg/mL; and (**F**) RES. Q1-LL represents normal cells, Q1-LR represents early apoptotic cells, Q1-UR represents late stage apoptotic cells, and Q1-UL represents necrotic cells. (**G**) Results are expressed as mean ± SEM (*n* = 3). * *p* < 0.05 vs. the control group, ^#^ *p* < 0.05 vs. 36 μg/mL of MC-LR group. (**H**) The effects of apoptosis on the testicular tissues of rats were tested by terminal deoxynucleotidyl transferase dUTP nick end labeling (TUNEL) assay and confocal microscopy. TUNEL assay was used to examine the apoptosis rate of testicular tissues in rats exposed to MC-LR with or without RES: green, TUNEL-positive cell; blue, nuclear. Left panel: at least 10 tubules in each random field and three fields randomly selected were evaluated for each testis (100×), Scale bar: 25 μm. Right panel: magnified images (300×) of the boxes in the left panel, scale bar: 50 μm. Three testes from different mice were tested in each group. (**I**) The results are expressed as mean ± SEM (*n* = 9); * *p* < 0.05 vs. the control group, ^#^ *p* < 0.05 vs. 40 μg/kg of the MC-LR group. PI: propidium iodide.

After 24 h of exposure to MC-LR in vitro, the co-cultured Sertoli–germ cell apoptosis rates which included early apoptosis and late apoptosis were examined by Annexin V–fluoresceine isothiocyanate/propidium iodide (FITC/PI) apoptosis detection kits. As shown in Figure 2A–G, the apoptosis significantly increased at 9 μg/mL or higher concentrations of MC-LR. However, the apoptosis rate was remarkably decreased when pretreatment with RES (20 μM) for two hours. Terminal deoxynucleotidyl transferase dUTP nick end labeling (TUNEL) tests and confocal microscopy were performed in vivo to detect the induction of apoptosis in the testicular tissues of rats exposed to MC-LR. As shown in Figure 2H,I, the expression of testicular apoptotic (TUNEL-positive) cells increased more than in the control group. However, pretreatment with RES for two hours followed by MC-LR injection dramatically alleviated the apoptotic cells in the testis when compared to the MC-LR group.

### 2.3. Effect of RES on MC-LR-Induced Mitochondrial Membrane Collapse 

**Figure 3:**
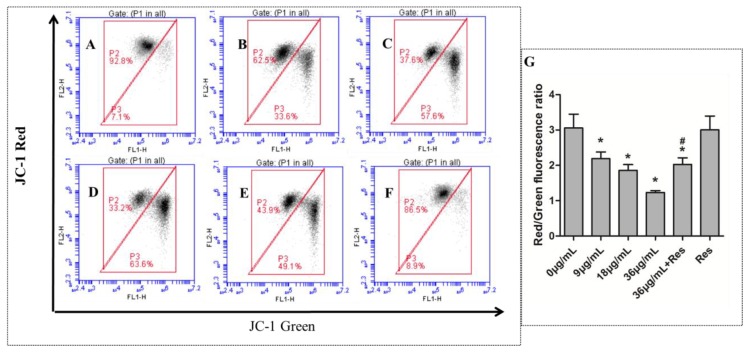
Effects of RES and MC-LR on the mitochondrial membrane potential in Sertoli–germ cells. The mitochondrial membrane potential of cells was detected by JC-1 staining and flow cytometry: (**A**) 0 μg/mL; (**B**) 9 μg/mL; (**C**) 18 μg/mL; (**D**) 36 μg/mL; (**E**) 36 µg/mL + RES; and (**F**) RES. (**G**) Quantitative analysis of green/red fluorescence in Sertoli–germ cells. Data are expressed as mean ± SEM (*n* = 3); * *p* < 0.05 vs. the control group, ^#^ *p* < 0.05 vs. the MC-LR group (36 μg/mL). Red/Green fluorescence ratio was calculated via mean red fluorescence value (mean FL2-H value) and mean green fluorescence value (mean FL1-H value) which were given by flow cytometer. P2: red fluorescent cells; P3: green fluorescent cells.

The early disruption of mitochondrial membrane potential (ΔΨm) is the most critical event during apoptosis, representing a prerequisite in drug-induced cell apoptosis. Therefore, in order to evaluate the effect of RES and MC-LR on the intrinsic pathway, the co-cultures of Sertoli–germ cells were labeled with JC-1 dye. MC-LR (9, 18, and 36 μg/mL) was found to induce mitochondrial membrane depolarization, characterized by the decrease in ΔΨ, as compared to the control group (Figure 3). Compared to the 36 μg/mL used in the MC-LR group, RES pretreated cells rescued the MC-LR-induced ΔΨ collapse. Thus, the observation confirmed that RES (20 μM) effectively mitigated the MC-LR-induced disruption of mitochondrial membrane potential (Figure 3). 

### 2.4. mRNA Levels of SIRT1/p53 Pathway Markers

**Figure 4:**
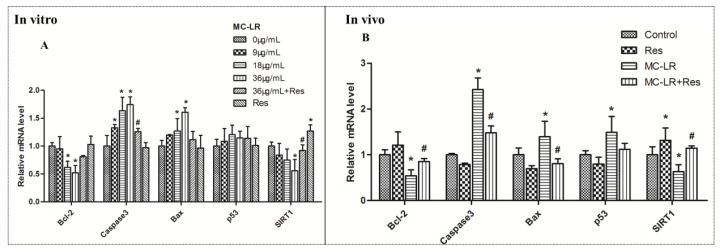
Apoptosis-related gene expression levels in co-cultured Sertoli–germ cells and testicular tissues exposed to MC-LR with or without RES. (**A**) Relative mRNA level in vitro; (**B**) Relative mRNA level in vivo. Data are presented as mean ± SEM for each group; * *p* < 0.05 vs. the control group, ^#^ *p* < 0.05 vs. the MC-LR group (in vitro: 36 μg/mL; in vivo: 40 μg/kg).

In order to examine whether MC-LR induced apoptosis through the *SIRT1*/*p53* pathway, and evaluate the effect of RES on the target genes, the effects of MC-LR on the mRNA levels of the *SIRT1*/*p53* pathway markers were tested. The co-culture of Sertoli–germ cells exposed to MC-LR (9, 18 and 36 μg/mL) with or without RES for 24 h and the mRNA expression of *SIRT1*, *p53*, *Bcl-2*, *caspase-3* and *Bax* were tested in vitro by qPCR analysis. MC-LR exposure significantly increased mRNA expression for *caspase-3* and *Bax* at 18 and 36 μg/mL in Figure 4A. A slight upregulation in *p53* mRNA expression was found in the MC-LR group, but there was no significant difference, when compared to the control group. Furthermore, *Bcl-2* and *SIRT1* mRNA expression was downregulated after exposure to 36 μg/mL of MC-LR. In the presence of 20 μM of RES, the MC-LR-induced upregulation of *caspase-3* and *Bax* was blunted. The suppression of *Bcl-2* and *SIRT1* mRNA expression by MC-LR was rescued by RES pretreatment (Figure 4A). MC-LR exposure in vivo induced the mRNA expression of *p53*, *Bax* and *caspase-3* to increase in testicular tissues (Figure 4B). Conversely, *SIRT1* and *Bcl-2* mRNA expression was decreased by MC-LR treatment. Compared to the MC-LR group, RES pretreatment suppressed the MC-LR-induced *p53*, *Bax*, and *caspase-3* mRNA expression, and increased *SIRT1* and *Bcl-2* mRNA levels, which was similar to the untreated group (Figure 4B). Taken together, these results indicate that RES could protect against MC-LR-induced germ cell apoptosis through the *SIRT1*/*p53* pathway. 

### 2.5. RES Increased SIRT1 Protein Expression and Mediated p53-Related Apoptotic Protein Expression

**Figure 5:**
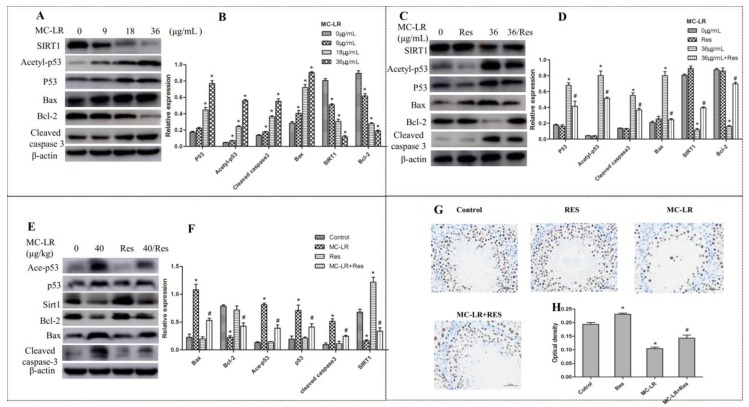
Apoptosis-related protein expression levels in co-cultured Sertoli–germ cells and testicular tissues exposed to MC-LR in the presence or absence of RES. (**A**–**D**) Western blot of sirtuin 1 (SIRT1), p53, acetylated p53, Bax, Bcl-2, and cleaved caspase-3 in co-cultured Sertoli–germ cells exposed to MC-LR in the presence or absence of RES; and (**E**,**F**) Western blot of apoptosis-related protein expression levels in testicular tissues of rats exposed to MC-LR with or without RES. The expression levels were quantified with Quantity One. β-actin was used as a loading control. Data are presented as mean ± SEM for each group; * *p* < 0.05 vs. the control group, ^#^ *p* < 0.05 vs. the MC-LR group (in vitro: 36 μg/mL; in vivo: 40 μg/kg). (**G**) Immunohistochemistry was used to examine the effect of MC-LR and RES on SIRT1 protein expression in rat testes (×400). Scale bar: 50 μm. (**H**) The quantitative analysis of SIRT1 expressed in each of the groups, * *p* < 0.05 vs. the control group, ^#^ *p* < 0.05 vs. the MC-LR group (40 μg/kg).

SIRT1 plays a number of crucial physiological roles in various cellular functions, such as gene silencing, cell cycle, apoptosis, and energy homeostasis [32]. Most notably, SIRT1 could deactivate p53 and attenuate its ability as a transcription factor, thereby ameliorating apoptosis [33]. In order to further explore the mechanisms of MC-LR-induced apoptosis, the protein expression of SIRT1, Acetyl-p53, Bax, Bcl-2, and cleaved caspase-3 were examined in the co-culture of Sertoli–germ cells and testicular tissues by western blot. The expression levels of p53, Acetyl-p53, cleaved caspase-3, and Bax in vitro exhibited an increase within the dose range of 9-36 μg/mL, and these levels peaked at the highest concentration of MC-LR (36 μg/mL) (Figure 5A). Conversely, with increasing doses of MC-LR, the protein levels of Bcl-2 and SIRT1 were significantly inhibited. Hence, 36 μg/mL of MC-LR had the most significant effect on Sertoli–germ cells, and was thereby used for subsequent experiments. The pretreatment with RES (20 μΜ) markedly rescued MC-LR-suppressed SIRT1 and Bcl-2 expression. In contrast, MC-LR (36 μg/mL) exposure dramatically increased p53, Acetyl-p53, cleaved caspase-3, and Bax protein expression, while RES pretreatment inhibited the induction effect of MC-LR (Figure 5C). The related protein expression levels in vivo were also examined to verify the mechanism of MC-LR-induced apoptosis. The SIRT1 protein level decreased in the MC-LR group as compared to the normal group, but a marked increase in the RES + MC-LR group was observed when compared to the former group. The acetylated p53, p53, cleaved caspase-3, and Bax protein levels were all significantly upregulated in the MC-LR-treated group, while RES pretreatment attenuated the MC-LR-induced protein changes. Similarly, the Bcl-2 protein level was reduced by MC-LR treatment, and was rescued by RES pretreatment (Figure 5E). In addition, the testicular SIRT1 expression decreased to approximately 50% in the MC-LR group, as compared to the control group (Figure 5G). However, the protein expression increased in RES-treated rats when compared to that in untreated rats. Furthermore, RES pretreatment with MC-LR injection improved the MC-LR-induced downregulation of SIRT1 expression. In summarize, the protective effect of RES on MC-LR-induced apoptosis may involve stimulation of SIRT1 followed by deactivation of p53 and regulation of apoptosis-related protein expression.

### 2.6. The Level of Acetylated Ku70 and Ku70–Bax Binding in the Co-Culture of Sertoli–Germ Cells and Testicular Tissues

**Figure 6:**
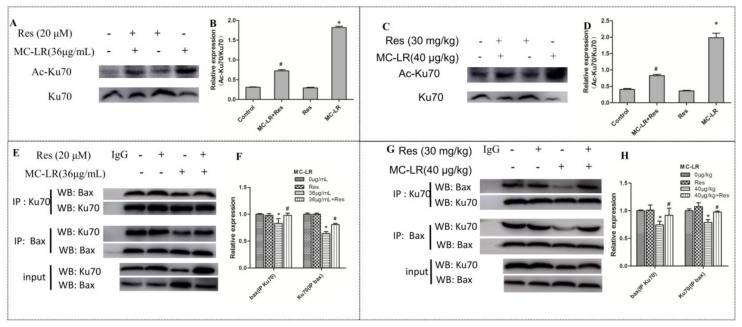
The western blot of acetylated Ku70 protein levels and the immunoprecipitation analysis of the Ku70–Bax binding state in co-cultures of Sertoli–germ cells and testicular tissues. (**A**,**B**) The western blot of acetylated Ku70 in co-cultures of Sertoli–germ cells after treatment with MC-LR with or without RES; (**C**,**D**) The western blot of acetylated Ku70 in the testicular tissues of rats exposed to MC-LR with or without RES; (**E**,**F**) The change in Ku70–Bax binding state in co-cultured Sertoli–germ cells exposed to MC-LR with or without RES by immunoprecipitation analysis; (**G**,**H**) The change in Ku70–Bax binding state in testicular tissues of rats exposed to MC-LR with or without RES by immunoprecipitation analysis. The band intensities of the immunoblots were quantified using Quantity One software, and are statistically presented in graphs. Data are presented as mean ± SEM for each group; * *p* < 0.05 vs. the control group, ^#^ *p* < 0.05 vs. the MC-LR group (in vitro: 36 μg/mL; in vivo: 40 μg/kg).

Ku70 is known to bind to Bax and isolate it from the mitochondria to regulate apoptosis [27]. Acetylation of Ku70 causes the isolation of Bax from Ku70 and accelerates cell death through Bax-mediated apoptosis. With the above results showing that the deacetylase SIRT1 was regulated after MC-LR or RES treatment, it was examined whether the change in SIRT1 could mediate Ku70 deacetylation in Sertoli–germ cells. As shown in Figure 6A,B, MC-LR significantly enhanced Ku70 acetylation in Sertoli–germ cells. Interestingly, pretreatment with RES lowered the content of acetylated Ku70, when compared to the MC-LR group. Moreover, co-immunoprecipitation was used to confirm the interaction between Bax and Ku70 (Figure 6E). These results show that even though the Ku70–Bax binding complex was disrupted by MC-LR, RES could ameliorate the disruption effects of MC-LR on the Ku70–Bax binding. This suggests that MC-LR disrupted the interaction of Ku70 with Bax, induced Ku70 acetylation and stimulated Bax dissociation from Ku70. Then, the dissociated Bax enters the mitochondria to trigger apoptosis and Sertoli–germ cell death.

Rats were exposed to MC-LR for 14 days in vivo, after which the acetylated Ku70 was found to be upregulated. The effect of RES combined with MC-LR treatment was reductive, decreasing total acetylation (Figure 6C). In order to determine whether Ku70 interacted with Bax induced by MC-LR in the testis, co-immunoprecipitation was used to assess the interaction of Ku70–Bax. As shown in Figure 6G,H, the treatment with MC-LR alone significantly decreased the content of Bax, which was connected to Ku70. In reverse-IP experiments, the amount of Ku70 was also lower than the control group. These results indicate that the Ku70–Bax interaction is disrupted by MC-LR treatment. RES-pretreated rats improved the disruptive effects of MC-LR on the interaction. Based on the above results, it was speculated that MC-LR disrupts the interaction of Ku70–Bax, stimulates the acetylation of Ku70 and releases Bax, allowing it to translocate to the mitochondria and thereby trigger apoptosis.

### 2.7. Effect of RES on MC-LR-Induced Pathological Change 

**Figure 7:**
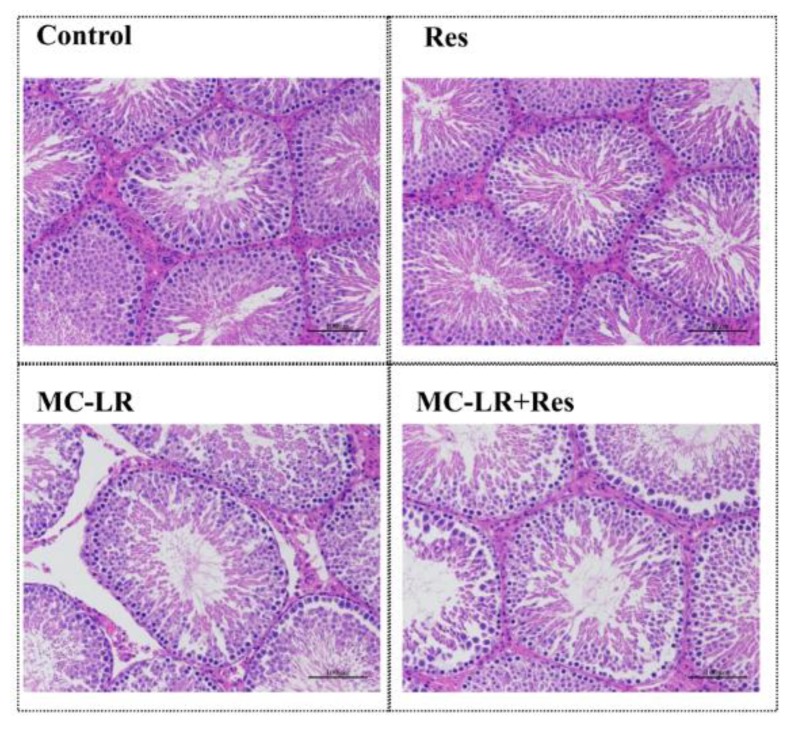
Effect of RES on MC-LR-induced pathological changes in the testis. The morphologic changes in testes exposed to MC-LR with or without RES (×200). The morphological changes of rat testes were examined by H&E staining. Scale bar: 100 μm.

In order to assess the effects of MC-LR and RES on the testis, the testicular histomorphology of SD rats was performed after MC-LR treatment for 14 days. The histopathological changes in the testis and seminiferous tubules were observed under a light microscope in different groups. MC-LR made the testis structure loose, and caused seminiferous tubule degeneration, structural shrinkage, and vacuolation in the mesenchyme. In RES-pretreated SD rats, Sertoli–germ cells were arranged in regular seminiferous tubules and compared to those of MC-LR-treated rats (Figure 7). 

## 3. Discussion

There is increasing evidence that infertility in animals and humans is potentially linked to environment exposure to reproductive toxins. Although we used a higher dose than that suggested by the WHO (1 μg/L), the concentration is usually much higher in natural water (10 μg/L). The highest microcystins contents in fish intestines were 85.67 μg/g dry weight (DW) [34]. The microcystins contents in cyanobacteria of water bloom can reach 7300 μg/g DW [35]. In addition, humans and animals can be exposed to microcystins in a variety of ways, such as through the digestive tract, respiratory tract, skin, and food chain [36]. Therefore, it is difficult for humans and animals to avoid the risk caused by higher doses of microcystins. The previous study conducted by the investigators demonstrated that MC-LR induced the apoptotic death of Sertoli cells and CHO cells through the activation of the mitochondrial caspase cascade [37], as well as ERs and autophagy [14], respectively. In the present study, a combination of in vitro and in vivo studies were used to investigate the mechanistic basis of the toxic effects of MC-LR on Sertoli–germ cells, and determine whether RES could rescue cells from apoptosis. 

The interactions between germ cells and Sertoli cells are crucial for the successful production of male gametes [38]. Interference in the normal interaction between Sertoli and germ cells may cause testicular dysfunction, which could result in the improper release of mature sperm [39]. The inhibited cell proliferation and upregulated cell apoptosis in Sertoli–germ cells isolated from rat testes by MC-LR indicated a detrimental effect on the male reproductive system. The polyphenol RES possesses comprehensive biochemical and physiological properties, ranging from antiplatelet, anti-inflammatory, and neuroprotective activity to anti-apoptosis [40]. In this study, RES pretreatment attenuated MC-LR-induced Sertoli–germ cell apoptosis and stimulated cell proliferation. The loss of mitochondrial membrane potential plays a vital role in the mitochondria-mediated apoptotic pathway. This study showed that MC-LR induced the depolarization of mitochondrial membrane potential, and caused ΔΨ collapse. However, RES pretreatment prevented the MC-LR-induced disruption of ΔΨ. Such data suggests that RES protected against MC-LR-induced ΔΨ collapse.

MC-LR induces reproductive toxicity by being transported into testicular tissues and stimulating cell apoptosis by targeting the spermatogonia and Sertoli cells [41]. SIRT1 is a nicotinamide adenine dinucleotide (NAD+)-dependent class III histone deacetylase, and has important physiological roles in regulating cell survival, and protecting against apoptosis [42]. In this study, SIRT1 expression was inhibited both in MC-LR-treated Sertoli–germ cells and co-cultured cells obtained from rats treated with MC-LR for 14 days. Moreover, MC-LR treatment promoted cellular apoptosis. RES is a SIRT1 activator [43] that can mitigate type-1 diabetes mellitus-induced sperm abnormalities and DNA damage by activating SIRT1 [44]. RES inhibited H_2_O_2_-induced apoptosis by activating SIRT1 and inhibiting p53 acetylation and caspase-3 activation [45]. In the present study, there was a remarkable recovery in SIRT1 level in RES-pretreated Sertoli–germ cells, as well as in the in vivo rat model. In addition, the protein level of Bcl-2 was significantly suppressed by the sole treatment with MC-LR for 24 h, while the p53 level, particularly acetylated p53, Bax and cleaved caspase-3, was promoted by the treatment with MC-LR. This indicates the regulatory effect of SIRT1 and mitochondria-dependence of MC-LR-induced apoptosis in vitro and in vivo. Interestingly, these present results also revealed that the pretreatment with SIRT1 activator RES inhibited the acetylation of p53 and alleviated the suppression of Bcl-2 in both Sertoli–germ cells and rat testes. Furthermore, MC-LR-induced caspase cleavage and Bax expression were inhibited, while Bcl-2 protein expression was upregulated by RES treatment. These results indicate that RES protected Sertoli–germ cells from MC-LR-induced apoptosis in vitro and in vivo through the upregulation of SIRT1 and deregulation of p53 acetylation.

Ku70 is an essential factor in the non-homologous end joining (NHEJ) pathway of DNA repair factor. Numerous previous studies have shown that Ku70 could bind to the apoptotic protein Bax to regulate cell death in the cytoplasm [26]. More importantly, SIRT1 can enhance DNA repair capacity by physically forming a complex with Ku70 and deacetylasing Ku70 in Q293A cells [30]. The study conducted by Anekonda et al. [46] revealed that RES can protect retinal cells from apoptosis through the downregulation of Bax, the upregulation of SIRT1 and Ku70 activity, and the inhibition of caspase-3 activity. The investigators suspected that SIRT1 activator RES might prevent against MC-LR-induced reproductive toxicity by deacetylation of Ku70. The present results revealed that the acetylated Ku70 and SIRT1 protein levels in the RES+MC-LR group were lower than those in the MC-LR group, both in vitro and in vivo. These results indicate that RES suppressed the acetylation of Ku70. In addition, Bax is member of the Bcl-2 protein family that promotes apoptosis. It can translocate to the mitochondria and promote mitochondrial membrane potential loss, subsequently leading to apoptosis. Ku70 can bind to the apoptotic protein Bax in the cytoplasm and block Bax-mediated cell death [28], while the acetylated form of Ku70 releases Bax, allowing it to translocate to the mitochondria, and trigger caspase-dependent apoptosis [29]. In another study, the treatment of A549 cells with epigallocatechin gallate-upregulated Ku70 acetylation blocked the combination of Ku70 and Bax, and subsequently triggered lung cancer cell apoptosis [47]. The present study revealed that Ku70 and the acetylated Ku70 may play a crucial role in the Bax-mediated mitochondrial apoptosis pathway. Furthermore, the present study found that after treating Sertoli–germ cells and rats with MC-LR, the interaction of Bax–Ku70 markedly decreased. Surprisingly, the combined treatment of RES and MC-LR reduced the acetylation of Ku70, resulting in the effective increase in the interaction of Bax–Ku70, ultimately protecting against MC-LR-induced Sertoli–germ cell apoptosis due to RES. The above results showed that RES protected MC-LR-induced Sertoli–germ cells and rat testes from apoptosis by promoting the interaction of Bax–Ku70.

**Figure 8:**
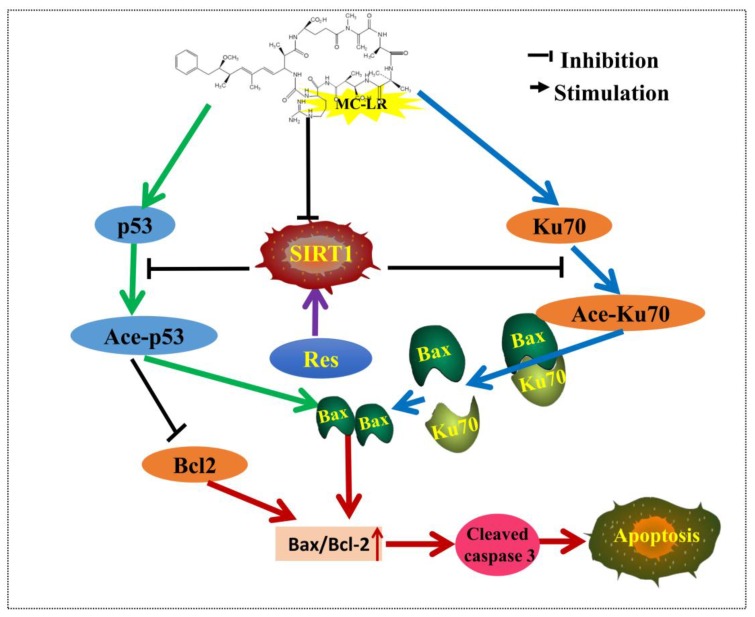
Proposed model for MC-LR-induced testis germ cell apoptosis in rats via SIRT1 signaling pathway activation. MC-LR exposure downregulated SIRT1 levels in primary co-cultured Sertoli–germ cells and rat testes, subsequently increasing p53 and Ku70 acetylation, and Bax/Bcl-2 and cleaved caspase-3 expression. RES pretreatment could promote the activation of SIRT1, which could resist MC-LR-induced apoptosis via reducing the acetylation of p53 and Ku70, as well as Bax/Bcl-2 and caspase-3 activation.

In conclusion, the present study revealed that MC-LR exposure downregulated SIRT1 levels in primary co-cultured Sertoli–germ cells and rat testes, which was accompanied by the upregulation of p53 and Ku70 acetylation, Bax expression, and cleaved caspase-3, and a decrease in Bcl-2 expression. RES pretreatment promoted the activation of SIRT1 and the interaction of Ku70 and Bax, and downregulated the acetylation of p53 and Ku70, Bax expression, and caspase-3 activation (Figure 8). This data suggests that the administration of RES ameliorates MC-LR-induced testis germ cell apoptosis, and protects against reproductive toxicity in rats by stimulating the SIRT1/p53 pathway. 

## 4. Materials and Methods 

### 4.1. Chemicals 

MC-LR (purity > 96%) was purchased from Beijing Express Technology Co. (Beijing, China). An institutional safety procedure was used to carry out the experiment according to the textbook of the “*Experimental methods and techniques of toxicology*”. Dulbecco’s modified eagle medium/nutrient mixture F-12 (DMEM/F-12), fetal bovine serum (FBS), penicillin–streptomycin, 0.25% trypsin, and collagenase type I were purchased from GIBCO (Rockville, MD, USA). RES was purchased from Abcam (Cambridge, UK). The annexin V–FITC/PI apoptosis detection kit and mitochondrial membrane potential assay kit were purchased from Beyotime Institute of Biotechnology (Shanghai, China). Cell Counting Kit-8 (CCK8) was purchased from Dojindo Laboratories (Kyushu Island, Japan). 

### 4.2. Isolation and Identification of Sertoli–Germ Cells

The isolation and identification of co-cultured Sertoli–germ cells were performed as previously other investigators described [48], with some improvements. Specific pathogen-free (SPF) male Sprague-Dawley (SD) rats (20–22 days old) were obtained from the Experimental Animal Center. Sertoli–germ cells were separated from these SD rats. Briefly, the testes were removed membrane, cut into pieces, washed two times with a pre-chilled PBS, and digested with 0.25% trypsin in an incubator for 30 min at 37 °C. Next, 0.1% collagenase was used to digest testicular fragments for another 30 min. Then, the homogenate was filtered by a 200-mesh stainless steel filter, and cells were washed with PBS for two times after collection. After centrifugation for five minutes at 1000 rpm, the cell pellets, which included the Sertoli cells and germ cells, were resuspended with DMEM/F-12 medium contained 10% FBS, and cultured in an incubator for 24 h at 37 °C (95% air and 5% CO_2_). Hematoxylin and eosin (H&E) staining was used to identify the co-cultured Sertoli–germ cells. The resuspended cells climbed to the carry sheet glass (20 mm), and were cultured for 24 h. The slides of these cells were cleaned using cold PBS, and fixed 15 min by 4% paraformaldehyd. Then, the cell specimens were assigned for H&E staining for routine histological examinations. The slides of these cells were examined under a microscope (Nikon Eclipse E100, Tokyo, Japan).

### 4.3. Cell Viability Assay

Sertoli–germ cells (density: 1 × 10^5^ cells per mL) were inoculated to a 96-well plate with 200 µL culture medium. After 24 h, cells were used with MC-LR for final concentrations of 0, 1, 5, 10, 20, 40, and 60 µg/mL for 24 h, and RES at final concentrations of 0, 1, 5, 10, 20, 30, 40, and 60 µmol/L for another 24 h. Next, CCK8 reagents were added to each well and incubated at 37 °C for four hours. Automated microplate reader (BioTek, Winooski, VT, USA) was used to measure absorbance at 450 nm. Cell inhibition rate and viability were calculated, and the IC50 dose of MC-LR for 24 h was determined. Cell viability = [(As − Ab)/(Ac − Ab)] × 100%, and inhibition rate = [(Ac − As)/(Ac − Ab)] × 100%. As: experimental hole absorbance (including medium, cells, CCK8, MC-LR, or RES), Ac: control hole absorbance (including medium, cells, CCK8, non-MC-LR, or RES), Ab: blank hole absorbance (including medium and CCK8, non-cells, non-MC-LR, or RES).

### 4.4. Apoptosis Assay

Flow cytometry was used to examine the apoptosis rate of Sertoli–germ cells. Briefly, cells were transplanted to a 6-well plate and exposed to different concentrations of MC-LR (0, 9, 18 and 36 µg/mL) with or without RES (20 µM). After 24 h, cells were collected, washed with cold PBS for two times and centrifuged at 1000 rpm for five minutes. Then, 5 × 10^5^ cells were selected for resuspension in 500 µL binding buffer, 5 µL annexin V-FITC, and 5 µL PI. These cells were kept in the dark at room temperature for 15 min. Flow cytometry was used to examined cells by a FACS Calibur flow cytometer (BD Accuri C6, Franklin Lakes, NJ, USA), and analyzed with the software. 

### 4.5. Measurement of Mitochondrial Membrane Potential 

JC-1, a cationic dye that accumulates in energized mitochondria, is an indicator of mitochondrial potential in a variety of cell types. Briefly, cells exposed to MC-LR with or without RES were collected and washed. Next, cells were loaded with 500 µL of JC-1 working solution. Then, these cells were incubated at 37 °C for 25 min. The fluorescence was detected with a FACS Calibur flow cytometer (BD Accuri C6, Franklin Lakes, NJ, USA).

### 4.6. Western Blot

Total protein was extracted from the testes of rats and co-cultured Sertoli–germ cells exposed to different concentrations of MC-LR with or without RES. Samples containing 30 µg of protein underwent electrophoresis with a Bio-Rad electrophoresis apparatus (Bio-Rad, Hercules, CA, USA), were separated by 12% SDS-PAGE, and subsequently transferred onto a polyvinylidene fluoride (PVDF) membrane (Millipore, Bedford, MA, USA). The membranes were blocked in tris buffered saline with tween (TBST) containing 5% BSA at 23 °C for two hours, and immunoblotted using primary anti-Ku70 (sc-17789, Santa Cruz Biotechnology, Boston, CA, USA), anti-SIRT1 (ab110304), anti-p53 (ab131442), anti-p53 (acetyl K381, ab61241), anti-cleaved-caspase-3 (ab2302), anti-Bax (ab32503), anti-Bcl-2 (ab7973), and anti-β-actin (ab6276) (Abcam, Cambridge, UK). An enhanced chemiluminescence detection kit (Beijing ComWin Biotech Co., Ltd., Beijing, China) was used to analyze the protein bands. The intensity of the bands was quantified using the Bio-Rad Quantity One software. 

### 4.7. Real-Time PCR 

Total RNA was isolated from cells and the testes of rats by TRIZOL reagent (TaKaRa, Dalian, China). The purity of the RNA was tested using the quotient of the optical density (OD) at 260/280 nm. Then, the purified total RNA (1 mg) was reverse-transcribed using an EasyScript First-Strand cDNA synthesis super mix kit (TaKaRa, Dalian, China). The qPCR analysis was performed using SYBR Premix Ex Taq II. The PCR reaction was performed at 95 °C (5 min), followed by 45 cycles of denaturation for 95 °C (15 s), 60 °C (20 s), and 72 °C (20 s). The 2-ΔΔCt method was used to calculate the transcriptional levels of genes, with glyceraldehyde-3phosphate dehydrogenase (GAPDH) as the internal reference. Each sample was run in triplicate for qPCR. The sequences of primer pairs used in this assay were in the Table 1.

### 4.8. Animal Treatment

SPF male SD rats were purchased from the Henan Province Experimental Animal Center (license number: SCXK (YU) 2015-0004) and were fed at the barrier environment animal laboratory of colleague of public health in Zhengzhou University (license number: SYXK (YU) 2012-0007). The animals were fed with standard rodent pellet diet, provided with water ad libitum, and kept on a 12-h light–dark cycle. Rats were handled according to the guidelines for the care and use of laboratory animals published by the Ministry of Health of the People’s Republic of China. All studies were approved by the Animal Study Committee of Zhengzhou University (Date: March, 2014). Twenty-four male SD rats were randomly divided into four groups: control, RES (30 mg/kg bw) [49], MC-LR (40 μg/kg·bw), and RES+MC-LR groups. Rats were treated daily with MC-LR or vehicle by i.p. injection for 14 days. The rats in the RES+MC-LR group were pretreated with RES for two hours prior to MC-LR injection. At 24 h after the last injection, the testes of rats were excised for analysis. 

### 4.9. Hematoxylin-Eosin Staining

The testes were quickly collected from the SD rats, cleaned using cold PBS, and fixed with 4% paraformaldehyde for 24 h. Then, 30% phosphate-buffered sucrose solution was used to equilibrate the testes for 2 h. Testes were embedded in paraffin and cut into 6-μm sections. Next, the sections were dehydrated by xylene and 100% alcohol, subsequently conducted hematoxylin staining and eosin staining. Finally, a microscope (Nikon Eclipse E100, Tokyo, Japan) was used to observe the morphological changes of testes.

### 4.10. TUNEL Assay

The terminal deoxynucleotidyl transferase dUTP nick end labeling (TUNEL) (Roche, Switzerland) was used to perform the detection of apoptosis. Briefly, the testes were fixed in 4% paraformaldehyde for 24 h at 23 °C, permeabilized with 0.1% Triton X-100, and washed twice. Then, the terminal deoxynucleotidyl transferase (TdT)-labelled nucleotide mix was added to each slide, incubated for one hour at 37 °C, and observed using a fluorescent microscope (Olympus, Tokyo, Japan) at 488-nm excitation and 530-nm emission. Image-Pro Plus 6.0 (Media Cybernetics, Inc., Rockville, MD, USA) was used to select the labeled green fluorescent nuclei as a unified standard for judging positive cells in all photographs. DAPI blue nuclei with the same markers were selected as the total cells. The percentage of positive cells (number of positive cells / number of total cell × 100) was determined as the apoptosis rate (%).

### 4.11. Histology and Immunohistochemistry

The testes obtained from rats were fixed in buffered 4% formaldehyde and embedded in paraffin. Immunohistochemistry was performed with paraffin sections (5 μM) obtained from the testes specimen. The slides were incubated with a diluted primary antibody, anti-SIRT1 (1:1000, Abcam), while negative control was incubated with antibody diluents. Horse radish peroxidase (HRP)-labeled secondary antibody (Beijing ComWin Biotech, Beijing, China) was added to the specimens, and incubated for 30 min at 23 °C. Then, the sections were counterstained with hematoxylin, subsequently dehydrated, and observed under a light microscope (XSP-C204, CIC). Image-Pro Plus 6.0 (Media Cybernetics, Inc., Rockville, MD, USA, 2006) was used to select the same brown color as a unified standard for judging the positive of all photographs. Each photograph was analyzed to quantify the integral optical density of the immunohistochemical staining of each photograph.

### 4.12. Co-Immunoprecipitation Analysis 

The testes were rapidly homogenized in Radio-Immunoprecipitation Assay (RIPA) buffer. The protein was precleared with Protein A agarose suspension (Abcam, Cambridge, UK), and 500 μg of protein were incubated with 5 μg of the antibody (Ku70/Bax) on a benchtop shaker for two hours at room temperature. After incubation, the mixture was rotated on a benchtop shaker by adding 100 μL of Protein A agarose suspension at 4 °C overnight. Beads were collected and washed with RIPA buffer. After that, beads were boiled for five minutes in 5 × SDS sample loading buffer, subsequently separated by 12% SDS-PAGE, and transferred onto PVDF membranes for western blot.

### 4.13. Statistical Analysis

Data are expressed as mean ± standard error of the mean (SEM). One-way analysis of variance (ANOVA, Birmingham, UK) was used to analyze the significant differences between groups, followed by Student–Newman–Keuls test. *p* < 0.05 was considered statistically significant. All statistical analyses were carried out using SPSS 21.0 (Armonk, NY, USA, 2012).

## Figures and Tables

**Table 1:** The sequences of primers used in Real-Time PCR

Gene	Forward Primer	Reverse Primer
*SIRT1*	5′-TCATTCTGACTGTGATGACGA-3′	5′-CTGCCACAGTGTCATATCCAA-3′
*P53*	5′-CCCCTGAAGACTGGATAACTGTC-3′	5′-AACTCTGCAACATCCTGGGG-3′
*Bax*	5′-GAACCATCATGGGCTGGACA-3′	5′-GTGAGTGAGGCAGTGAGGAC-3′
*Bcl-2*	5′-CTGAACCGGCATCTGCACAC-3′	5′-GCAGGTCTGCTGACCTCACT-3′
*Caspase-3*	5′-GACTGCGGTATTGAGACAGA-3′	5′-CGAGTGAGGATGTGCATGAA-3′
*GAPDH*	5′-GGCACAGTCAAGGCTGAGAATG-3′	5′-ATGGTGGTGAAGACGCCAGTA-3′
